# Effects of combined β-hydroxy-β-methylbutyrate (HMB) and whey protein ingestion on symptoms of eccentric exercise-induced muscle damage

**DOI:** 10.1186/s12970-016-0119-x

**Published:** 2016-02-29

**Authors:** Minayuki Shirato, Yosuke Tsuchiya, Teruyuki Sato, Saki Hamano, Takeshi Gushiken, Naoto Kimura, Eisuke Ochi

**Affiliations:** Department of Hygiene and Public Health, Nippon Sport Science University, 7-1-1 Fukasawa, Setagaya, Tokyo, Japan; Laboratory of Health and Sports Sciences, Meiji Gakuin University, 1518 Kamikurata, Totsuka, Yokohama, Kanagawa Japan; The Research Institute of Nippon Sport Science University, 7-1-1 Fukasawa, Setagaya, Tokyo, Japan; Graduate School of Education, Okayama University, 3-1-1 Tsushimanaka, Kita, Okayama, Japan

**Keywords:** Amino acids, Muscle strength, Lengthening, Muscle soreness

## Abstract

**Background:**

The purpose of this study was to examine the effects of combined β-hydroxy-β-methylbutyrate (HMB) and whey protein ingestion on muscle strength and damage following a single bout of eccentric exercise.

**Methods:**

Eighteen untrained male subjects were assigned to HMB and Whey protein (HMB + Whey; 3 g/day HMB and 36.6 g/day whey protein, *n* = 6), HMB (3 g/day, *n* = 6), or whey protein (36.6 g/day, *n* = 6) groups. Ingestion commenced 7 days before non-dominant elbow flexor eccentric exercise (30 deg/sec, 6 reps × 7 sets) and continued until 4 days post-exercise. The maximal isometric strength, muscle soreness, plasma creatine kinase (CK), lactate dehydrogenase (LDH) were assessed pre-exercise, and at 1, 2, 3, and 5 days after exercise.

**Results:**

The change scores of maximal isometric strength significantly decreased at day 1, 2, and 5 in the whey protein group compared to pre value and that in HMB + Whey protein and HMB groups decreased at day 1 and 5. The muscle soreness significantly increased in the whey and HMB + Whey protein groups at day 3 compared to pre value (*p* < 0.05). CK and LDH significantly increased (time effect: *p* < 0.05) after exercise. However, all data were not significant difference among the groups.

**Conclusions:**

These results suggest that ingestion of combined HMB and whey protein does not have a role to inhibit muscle strength loss and soreness, and decrease in muscle damage markers after eccentric exercise in comparison with HMB and whey protein alone.

## Background

High-intensity resistance training involving eccentric contractions appears essential for increasing muscular strength and hypertrophy. However, such intense exercise disrupts the microstructure of muscle fibers and can result in severe muscle soreness, reduced strength, and reduced overall performance. Therefore, minimizing muscle soreness and enhancing muscle recovery may optimize strength and hypertrophy after exercise sessions. One common strategy to optimize recovery is nutritional supplementation. The effects of ingesting protein [1, 2], essential amino acids [3], and branched-chain amino acids [4, 5] as supplements on reduce muscle strength loss and soreness after resistance training and to promote muscle hypertrophy have been investigated.

In particular, whey protein isolate with high protein purity contains abundant essential and branched-chain amino acids [6] and has rapid absorption kinetics than casein protein [7]. Whey protein isolate ingestion increases blood amino acid concentration and stimulates muscle protein synthesis [6]. Ingestion of whey protein isolate of 15 g has been reported to enhance signaling for muscle protein synthesis through mammalian target of rapamycin (mTOR) [8]. The ingestion of whey protein isolate in conjunction with resistance training significantly increased the cross-sectional area and muscle strength in previous studies [9, 10]. In the short-term study, compared with carbohydrate ingestion, whey protein isolate ingestion (1.5 g/kg/day) during 14-day eccentric contraction exercise of the leg increased the isometric muscle strength of knee extension and tended to lower plasma lactate dehydrogenase (LDH) levels [11]. However, White et al. [12] observed that eccentric contraction exercise of the legs had no effect on muscle strength and damage markers when they investigated the short-term effect of simultaneous ingestion of 23 g of whey protein and 75 g of carbohydrate. It suggests that the short-term effects of whey protein isolate ingestion have not been clearly demonstrated in human studies.

Meanwhile, the effects of ingestion of the amino acid metabolite β-hydroxy-β-methylbutyrate (HMB) in combination with resistance training have been studied in terms of enhancing recovery, strength, and muscle soreness [13]. HMB is a branched-chain amino acid leucine metabolite. Leucine is metabolized to α-ketoisocaproate (KIC) by the enzyme branched-chain amino acid transferase KIC metabolized to HMB in cytosol [14, 15]. HMB has been reported to be involved in the supply of the main component of the muscle cell membrane, cholesterol [16], and signaling for muscle protein synthesis through the mTOR [17]. Nissen et al. [18] and Panton et al. [19] reported that HMB ingestion (3 g/day) during 3–4 weeks of resistance training decreased creatine kinase (CK). In the short-term study, 14-day HMB ingestion (3 g/day) inhibited an increase in CK and muscle soreness after exercise involving eccentric contractions of the elbow [20]. However, Paddon-Jones et al. [21] observed that 24 eccentric contractions of the elbow flexor had no effect on muscle strength and soreness in a study in which HMB was ingested (40 mg/[kg⋅day]) for 16 days, including a 6-day prior ingestion. Nunan et al. [22] observed no effect on muscle strength and CK after downhill running when HMB and KIC (HMB: 3 g/day, KIC: 0.3 g/day) were simultaneously ingested for 14 days, including an 11-day prior ingestion. Hence, the short-term effects of HMB ingestion also have not been clarified.

Based on these findings, we thought that the simultaneous ingestion of HMB and whey protein isolate synergistically promotes muscle protein synthesis and influences on muscle cellular function and net protein balance, accordingly it helps to prevent muscle strength loss, and reduces muscle damage after high-intensity eccentric exercise. However, the short-term effects of the combination of HMB and whey protein isolate ingestions have not been reported yet in previous studies.

Therefore, we investigated the effect of simultaneous ingestion of HMB and whey protein isolate on muscle strength and damage in the recovery process after transient eccentric contractions. The hypothesis was that ingestion of the combination of HMB and whey protein isolate would provide greater benefits than HMB or whey protein isolate alone during the recovery phase.

## Methods

### Subjects

This study was approved by the Ethics Committee of Nippon Sport Science University (ID: 012-H50). All participants were informed verbally, as to the aim and the potential risks of the experiments and provided written informed consent before starting this study, which complied with the Declaration of Helsinki. A total of 18 males aged 19.2 ± 0.4 years, 64.9 ± 7.3 kg, 170.9 ± 5.6 cm tall, and 12.5 ± 3.4 % body fat, took part in the study. Subjects were recreationally active (moderate exercise 4–5 times a week) and considered apparently healthy without a history of disease or medication use. Subjects reported that they had not engaged in any intense training, including eccentric contractions, sufficient to cause significant muscle soreness in the 3 months prior to experiment.

### Experimental procedure

**Fig. 1:**
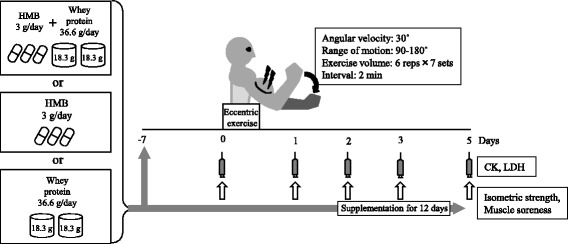
Experimental protocol. Ingestion groups: HMB (3 g/day) and whey protein (36.6 g/day) group, HMB (3 g/day) group, and whey protein (36.6 g/day) group. Supplements were taken for 7 days before and 4 days after eccentric exercise (supplementation for 12 days). Blood was analyzed before and at 1, 2, 3, and 5 days post-exercise. CK: creatine kinase, LDH: lactate dehydrogenase, Isometric strength: maximal voluntary isometric contraction torque of elbow in flexed position (90°), Muscle soreness: 100-mm visual analogue scale for biceps brachii

The subjects started supplement ingestion one week before the exercise experiment, and continued it until 4 days after the exercise, i.e., the duration of the ingestion period was 12 days in total. On the day of exercise, after body composition, blood sampling, subjective muscle soreness (Visual Analogue Scale: VAS) of the biceps brachii of the non-dominant arm, and maximum isometric muscle strength of the elbow were measured, followed by eccentric contraction exercise of the same arm. Blood analysis, VAS, and maximum isometric muscle strength of elbow flexion were performed 1, 2, 3, and 5 days after exercise (Fig. 1). Vigorous exercise causing muscle soreness, icing, massage, and ingestion of other supplements were prohibited throughout the experimental period.

### Supplements

The 18 subjects were randomly divided into 3 groups: groups for simultaneous ingestion of HMB and whey protein isolate (HMB + Whey group: n = 6, age: 19.0 ± 0.0 years, height: 168.6 ± 6.2 cm, weight: 60.7 ± 7.1 kg, body fat: 11.2 ± 3.3 %, lean body mass: 53.8 ± 5.5 kg), HMB alone (HMB group: n = 6, age: 19.3 ± 0.5 years, height: 170.6 ± 3.9 cm, weight: 67.3 ± 7.9 kg, body fat: 13.6 ± 3.7 %, lean body mass: 58.0 ± 5.1 kg), and whey protein isolate alone (Whey group: n = 6, age: 19.3 ± 0.5 years, height: 173.4 ± 6.2 cm, weight: 66.7 ± 6.2 kg, body fat: 12.7 ± 3.3 %, lean body mass: 58.1 ± 4.5 kg). The experiment was performed employing the double-blind method. For HMB, calcium-HMB capsules containing 1 g of HMB per capsule were ingested (Optimum Nutrition, Inc., USA): One capsule was ingested with water 3 times a day after breakfast, lunch, and supper (HMB: 3 g/day). For whey protein, whey protein isolate (ISO PRO, Bulk Sports, Japan) was ingested. A spoonful of whey protein (21 g, energy: 79 kcal, protein: 18.3 g, fat: 0.3 g, carbohydrate: 1.0 g, and sodium: 147 mg) mixed with 200 ml of water was ingested twice a day, after breakfast and supper (Whey protein isolate: 36.6 g/day). The ingredient percentage of essential amino acid and branched-amino acid in the whey protein isolate were 45.3 and 22.1 %, respectively.

### Eccentric exercise

Eccentric exercise was performed using the Biodex System 3 (Biodex Medical Systems, Inc., USA). The subject sat on the measurement device and placed the upper arm on the pad so as to set the shoulder joint of the non-dominant arm at 45° flexion. After adjusting the rotation axes of the elbow joint and dynamometer to the same level, the chest and lumbar region, and upper arm of the subject were fixed with straps. The subject then grasped and adjusted the moment arm to a length giving no discomfort to the wrist joint at 90 and 180° of the elbow joint angles. In the eccentric exercise of the elbow, eccentric contraction was repeated 6 times with maximum effort as one set, and 7 sets were performed at 2-min intervals (42 eccentric contractions in total, angular velocity: 30°/sec, range of motion: 90–180°).

### Measurement

Body mass and body composition were measured using a Tanita MC-190 multi-frequency body composition monitor (TANITA Co., Japan). Blood samples (~10 ml) were taken from the median antebrachial vein, and serum was separated by centrifugation (3000 rpm, 10 min). CK and LDH levels were measured in duplicate at each time point using a Japan Society of Clinical Chemistry Standardized Method [23], and the intra- and inter-assay CVs were 1.0 and 0.8 %, 0.9 and 0.8 %, respectively. Muscle soreness was evaluated using VAS, which is employed in various studies as a method to evaluate subjective muscle soreness [3, 5, 20, 21, 24]. A 100-mm scale from ‘no pain’ at 0 mm to ‘worst imaginable pain’ at 100 mm was set. Before measurement, the same examiner lightly pressed the subject’s biceps brachii at 7 cm above the elbow joint with a finger, and the subject marked his subjective muscle soreness on the scale. The measurement of the maximum isometric muscle strength of elbow flexion, the maximum static muscle strength of the non-dominant arm at an elbow joint angle of 90° was measured using Biodex System 3. Maximum contraction for 3 s was performed 3 times at 5-s resting intervals, and the highest value was adopted.

### Statistical analysis

All data are presented as the mean ± confidence interval (CI) (except physical characteristics data: mean ± SD). Measurements data were converted to change scores, compared to pre value (Maximum isometric muscle strength values were converted to those per body weight). The physical characteristics and total load of eccentric contraction were analyzed using one-way ANOVA, and the other measurements were analyzed using two-way ANOVA with ingestion groups and time (group vs. time). When a main effect was detected, Tukey HSD multiple comparison was performed. The significance level was set at p less than 5 %. For the statistical analysis software, SPSS ver.20.0 Advanced Statistics (IBM, Co., USA) was used.

## Results

### Isometric muscle strength

The change scores of maximum isometric muscle strength (per body weight) are presented in Table 1. Muscle strength was demonstrated a decrease after the eccentric exercise (time: *p* = 0.0001), but no group (*p* = 0.73) or group × time interaction (*p* = 0.90) was observed for each ingestion. All groups were significantly decreased on day 1 (Whey: *p* = 0.005, HMB + Whey: *p* = 0.03, HMB: *p* = 0.01) and day 5 (Whey: *p* = 0.006, HMB + Whey: *p* = 0.01, HMB: *p* = 0.02) compared to pre value. Only the Whey group was significantly decreased on day 2 (*p* = 0.04) compared to pre value. The mean percentage of muscle strength loss in all groups was showed −36.7 ± 4.3 % at day 1, after that it was slightly increased at day 2 (−24.2 ± 7.5 %) and day3 (−23.9 ± 3.4 %), but it was decreased at day 5 (−33.3 ± 1.0 %) compared to the pre values.

**Table 1:** Changes scores from pre-value in maximal voluntary isometric contraction (MVC) torque 1, 2, 3, 5 days after eccentric contractions in Whey, HMB + Whey, and HMB

MVC (Nm/kg)	Day 1	Day 2	Day 3	Day 5
Whey	−0.44 ± 0.28^**^	−0.37 ± 0.29^*^	−0.34 ± 0.41	−0.38 ± 0.21^**^
HMB + Whey	−0.35 ± 0.27^*^	−0.20 ± 0.32	−0.24 ± 0.24	−0.36 ± 0.24^*^
HMB	−0.40 ± 0.21^*^	−0.23 ± 0.22	−0.23 ± 0.20	−0.33 ± 0.23^*^

mean ± 95 % CI

** (*p* < 0.01), * (*p* < 0.05); a significant difference from pre-exercise value

### Muscle soreness

The change scores of muscle soreness on VAS are presented in Table 2. VAS was demonstrated a increase after the eccentric exercise (time: *p* = 0.0001), but no group (*p* = 0.93) or group × time interaction (*p* = 0.68) was observed for each ingestion. Whey group was significantly increased on day3 (*p* = 0.003) compared to pre value. HMB group was no significantly increased after eccentric exercise, but tended to increase on day 1 and day 2 (*p* = 0.08, *p* = 0.09, respectively). HMB + Whey group was significantly increased on day 3 compared to pre value (*p* = 0.03). Also, there were no significant differences in the total load of eccentric contraction among groups (HMB + Whey: 2.1 ± 0.5 kJ, HMB: 2.1 ± 0.6 kJ, Whey: 2.2 ± 0.5 kJ).

**Table 2:** Changes scores from pre-value in muscle soreness assessed by visual analog scale (VAS) 1, 2, 3, 5 days after eccentric contractions in Whey, HMB + Whey, and HMB

VAS (cm)	Day 1	Day 2	Day 3	Day 5
Whey	3.1 ± 4.5	4.0 ± 4.4	4.3 ± 2.9^**^	1.6 ± 3.2
HMB + Whey	2.9 ± 1.3	3.4 ± 1.6	3.3 ± 1.2^*^	1.3 ± 1.6
HMB	4.0 ± 3.5	4.0 ± 3.5	2.3 ± 2.7	1.0 ± 3.4

mean ± 95 % CI

** (*p* < 0.01), * (*p* < 0.05); a significant difference from pre-exercise value

### Muscle damage markers

The change scores of muscle damage markers are presented in Table 3. CK was demonstrated a increase after the eccentric exercise (time: *p* = 0.04), but no group (*p* = 0.38) or group × time interaction (*p* = 0.39) was observed for each ingestion. LDH was demonstrated an increase after the eccentric exercise (time: *p* = 0.04), but no group (*p* = 0.51) or group × time interaction (*p* = 0.55) was observed for each ingestion.

**Table 3:** Changes scores from pre-value in creatine kinase (CK) and lactate dehydrogenase (LDH) 1, 2, 3, 5 days after eccentric contractions in Whey, HMB + Whey, and HMB

	Day 1	Day 2	Day 3	Day 5
CK (U/L)				
Whey	−30.7 ± 512.4	1243.5 ± 2907.9	5946.2 ± 10557.6	6883.0 ± 12062.5
HMB + Whey	−152.3 ± 445.4	−186.2 ± 833.6	921.5 ± 3005.0	1541.2 ± 3572.7
HMB	10.3 ± 96.4	169.5 ± 683.1	1637.2 ± 2056.2	3093.2 ± 2585.7
LDH (U/L)				
Whey	−19.8 ± 32.0	20.2 ± 118.0	205.5 ± 444.6	216.5 ± 423.1
HMB + Whey	−23.0 ± 355.5	−29.5 ± 49.2	30.0 ± 226.3	60.0 ± 200.1
HMB	−27.5 ± 7.7	−21.0 ± 30.8	61.2 ± 175.9	112.0 ± 134.6

mean ± 95 % CI

### Discussion

The purpose of this study was to examine the effects of combined HMB and whey protein ingestion on muscle function and muscle damage markers after an acute bout of eccentric exercise. Our results demonstrate that simultaneous ingestion of HMB and whey protein isolate did not reduce muscle strength loss, muscle soreness or increase muscle damage markers after intense exercise in comparison with ingestion of HMB and whey protein isolate alone.

In the present study, a significant decrease in isometric muscle strength was noted on days 1 and 2 after exercise in the whey group, but the muscle strength loss on day 2 was inhibited in the HMB + Whey and HMB groups. Although not directly demonstrated in the present study because of the absence of a placebo group, these results suggest that simultaneous ingestion of HMB and whey protein is no effect for the reduction of muscle strength loss. Paddon-Jones et al. [21] reported that the ingestion of 40 mg/(kg⋅day) of HMB was ineffective for maximal isometric strength after 24 eccentric exercises and reported a percentage decrement in muscle strength similar to that in our study. Nosaka et al. [24] reported that maximal isometric strength loss was −45 % at day 1 and −37 % at day 4 after 24 maximal eccentric exercises on the elbow flexors. In our recent study, the significant muscle strength loss continued until 3 days after 30 eccentric elbow contractions were performed [25]. In the present study, the percentage of the baseline value of maximal isometric strength loss in all of the groups was −37 % at day 1, −24 % at days 2 and 3, −33 % at day 5 after 42 maximum eccentric contractions. These decrement rates in muscle strength from day 1 to 3 were lower in all of the groups in this study than in those in the previous study [24]. However, we have to notice that the reduction of muscle strength is still observed in 5 days after exercise. As the reason for the decrease in muscle strength at day 5, we suggest the excessive load of eccentric exercise. Although previous studies used maximum 12-30 eccentric contractions and confirmed the recovery until 4 days [26, 27], present study was 42 times with maximum efforts. In this point of view, we should monitor 3 or 5 more days to confirm the recovery phase.

Muscle soreness as assessed by the VAS significantly increased from its pre value in the HMB + Whey and Whey groups only at day 3 after eccentric exercise. This result suggests that no attenuating effect on muscle soreness occurred in all the groups. Paddon-Jones et al. reported that HMB ingestion had no beneficial effect on muscle soreness [21]. By contrast, Jackman et al. [28] and Nosaka et al. [3] observed a reduction in muscle soreness on the VAS by ingestions of branched-chain amino acid and a mixture of amino acids. Shimomura et al. [5] also reported that the ingestion of 5 g of branched-chain amino acids was effective for the relief of muscle soreness. In our study, the branched-chain amino acid content of a single dose of whey protein was about 4 g, which is smaller than the effective dose (5 g). Thus, no significant effect on muscle soreness was noted in all the groups.

While CK and LDH significantly increased until day 5 (time effect *p* < 0.05), change scores compared to pre value were no significant differences among the groups. Similar to the muscle strength, the synergistic effects of HMB and whey protein ingestion on muscle strength recovery after heavy eccentric exercise were difficult to observe. In comparison with the CK, LDH, and muscle strength, muscle soreness increased on day 3 in Whey and HMB + Whey, and returned to the baseline on days 5 in all groups This observation was independent from muscle damage markers and muscle strength loss. Nosaka et al. reported that increase in CK after eccentric contractions did not correlated muscle soreness [29]. Also, it has been shown that there is no relationship between muscle soreness and reduction of strength [27]. Further studies are required to investigate the relationships between muscle soreness and muscle strength, CK, and LDH on muscle damage by eccentric contractions.

Our research has certain limitations. First, we did not set the placebo group in present study. It was critical and we could not observe any significant differences among the groups in all measurements. The lack of placebo group that removal the effect of HMB and amino acids made the establishment of our hypothesis difficult in our study. Second, subjects were recreationally active not untrained. According to the position stand on HMB in International Society of Sports Nutrition, HMB appears most beneficial for untrained individuals [13]. Third, we did not consider ingestion timing and the duration before and after eccentric exercise. HMB ingestion for 2 weeks prior to an exercise bout was recommended and seemed to prevent muscle damage [13]. In the present study, the ingested duration was only 1 week prior to exercise. Moreover, plasma HMB concentration appears to peak 60–120 min after ingestion [13]. Wilson et al. [30] suggested that ingestion of HMB before exercise seemed to prevent increases in LDH. However, we did not regulate ingestion timing in detail during eccentric exercise. Further investigations in consideration of the placebo setting, ingestion timing, ingestion duration, and untrained subjects are necessary.

## Conclusions

We conclude that the simultaneous ingestion of HMB and whey protein could not inhibit muscle strength loss, increment of muscle damage markers after eccentric exercise, compare to ingestion of HMB and whey protein alone. However, our numbers of each ingestion groups were small size, and there were not significant difference among the groups for scattering of all measurements.
